# Assessing the severity of sleep apnea syndrome based on ballistocardiogram

**DOI:** 10.1371/journal.pone.0175351

**Published:** 2017-04-26

**Authors:** Zhu Wang, Xingshe Zhou, Weichao Zhao, Fan Liu, Hongbo Ni, Zhiwen Yu

**Affiliations:** School of Computer Science, Northwestern Polytechnical University, Xi’an, Shaanxi, China; University of Rome Tor Vergata, ITALY

## Abstract

**Background:**

Sleep Apnea Syndrome (SAS) is a common sleep-related breathing disorder, which affects about 4-7% males and 2-4% females all around the world. Different approaches have been adopted to diagnose SAS and measure its severity, including the gold standard Polysomnography (PSG) in sleep study field as well as several alternative techniques such as single-channel ECG, pulse oximeter and so on. However, many shortcomings still limit their generalization in home environment. In this study, we aim to propose an efficient approach to automatically assess the severity of sleep apnea syndrome based on the ballistocardiogram (BCG) signal, which is non-intrusive and suitable for in home environment.

**Methods:**

We develop an unobtrusive sleep monitoring system to capture the BCG signals, based on which we put forward a three-stage sleep apnea syndrome severity assessment framework, i.e., data preprocessing, sleep-related breathing events (SBEs) detection, and sleep apnea syndrome severity evaluation. First, in the data preprocessing stage, to overcome the limits of BCG signals (e.g., low precision and reliability), we utilize wavelet decomposition to obtain the outline information of heartbeats, and apply a RR correction algorithm to handle missing or spurious RR intervals. Afterwards, in the event detection stage, we propose an automatic sleep-related breathing event detection algorithm named Physio_ICSS based on the iterative cumulative sums of squares (i.e., the ICSS algorithm), which is originally used to detect structural breakpoints in a time series. In particular, to efficiently detect sleep-related breathing events in the obtained time series of RR intervals, the proposed algorithm not only explores the practical factors of sleep-related breathing events (e.g., the limit of lasting duration and possible occurrence sleep stages) but also overcomes the event segmentation issue (e.g., equal-length segmentation method might divide one sleep-related breathing event into different fragments and lead to incorrect results) of existing approaches. Finally, by fusing features extracted from multiple domains, we can identify sleep-related breathing events and assess the severity level of sleep apnea syndrome effectively.

**Conclusions:**

Experimental results on 136 individuals of different sleep apnea syndrome severities validate the effectiveness of the proposed framework, with the accuracy of 94.12% (128/136).

## Introduction

Sleep Apnea Syndrome is a common sleep-related breathing disorder, which is usually accompanied by partial or complete respiration cessation during night sleep. As it’s reported, the prevalence of sleep apnea syndrome is around 4% to 7% in male, and 2% to 4% in female population all around the world [1–3]. Generally, compared with subjects without sleep apnea syndromes, sleep apnea syndrome patients are more likely to suffer cardiovascular problems, such as hypertension, stroke and heart failure [4]. Moreover, severe sleep apnea syndrome may even result in decreased memory and cognitive decline [5].

Specifically, there are two main kinds of sleep-related breathing events when sleep apnea syndrome happens, i.e., the Apnea Event and the Hypopnoea Event [6]. The Apnea Event is defined as airflow cessation at nose and mouth, which lasts for at least 10 seconds. The Hypopnoea Event is defined as reduced airflow, which reduces more than 50% of the baseline for at least 10 seconds, and a fall in blood oxygen saturation (SpO_2_) at least 4% [7]. The Apnea Hypopnoea Index (AHI), which refers to the average number of apnea and hypopnoea per hour during night sleep, is utilized for medical specialists to diagnose sleep apnea syndrome and its severity. In particular, according to the American Academy of Sleep Medicine, subjects whose AHI < 5, 5 ≤ AHI < 15, 15 ≤ AHI < 30 and AHI ≥ 30 are regarded as healthy ones, mild sleep apnea syndrome suffers, moderate sleep apnea syndrome suffers and severe sleep apnea syndrome suffers [8], respectively.

Currently, the primary method to diagnose sleep apnea syndrome and measure its severity is using Polysomnography [9], which is the gold standard in sleep study field. PSG can record the user’s EEG (Electroencephalogram), EMG (Electromyography), EOG (Electro-Oculogram), SpO_2_, respiration effort, snoring and other physiological signals simultaneously in a sleep center. Based on these signals, sleep experts could diagnose sleep apnea syndrome and its severity manually. While PSG can provide comprehensive and objective physiological information about the user’s health status, many shortcomings still limit its generalization in home environment. First, during the signal recording procedure, users have to attach numerous electrodes on their bodies, which undoubtedly disturb their normal sleep, and even make them unable to fall asleep. Additionally, the PSG device is expensive and time consuming, which makes it unsuitable for home use [10, 11].

Due to the shortcomings of PSG, several alternative techniques have been developed to simplify the measurement of sleep apnea syndrome, such as single-channel ECG, pulse oximeter, ballistocardiogram (BCG) [12, 13], polyvinylidene fluoride sensors [14–17], acoustic sensors [18] and so on. Generally speaking, existing studies on sleep apnea syndrome assessment can be divided into two main categories from the methodology view, namely the direct approach and the indirect approach.

The direct approach conducts sleep apnea syndrome severity assessment based on the differences in various signals. These studies directly construct the severity evaluation models by analyzing and extracting features for each severity level, including mild, moderate, and severe. There are many practical assessment methods, including heart rate variability (HRV) analysis, entropy analysis and speech analysis. For example, [19–21] utilized the heart rate variability analysis, entropy analysis and speech analysis respectively, to analyze the ECG, SpO_2_ and voice signals individually other than all signals collected by PSG. Meanwhile, various features including time domain features, frequency domain features, and other features, are extracted to characterize different severity levels, and with the employment of different classifiers, the final severity states are obtained. However, as the differences between mild sleep apnea syndrome suffers and healthy subjects are not very significant [20, 21], these direct approaches can’t effectively distinguish all the severity levels.

The indirect approaches focus on detecting sleep-related breathing events firstly, and then calculating AHI to evaluate the final severity condition. In fact, most existing studies take the detection of sleep-related breathing events as a channel, and convert the original severity assessment problem into the sleep-related breathing event detection problem. Therefore, the indirect approach avoids the issue to directly distinguish mild sleep apnea syndrome suffers and healthy subjects. In general, there are two lines of methods for the detection of sleep-related breathing events. The first line of methods adopt the equal-length fragmentation approach, which divide physiological signals into a set of equal-length fragments, and then detect whether there are sleep-related breathing events in the fragments. Once a sleep-related breathing event is detected within a certain fragment, the equal-length fragmentation approach will take the whole fragment as the length of the event, which is a little far-fetched. In [22, 23], authors adopt 1min and 10s equal-length segmentations respectively to detect sleep-related breathing events, and they concentrate on the variation of the ECG signal and respiratory signal when sleep apnea syndrome happens. However, the equal-length segmentation method might divide one sleep-related breathing event into different fragments, leading to incorrect detection results. The other line of strategies is based on the unequal-length segmentation idea, which overcomes the event segmentation issue and can detect sleep-related breathing events more effectively [24, 25]. However, existing studies have not fully considered the practical factors of sleep-related breathing events, such as the limit of lasting duration, possible occurrence sleep stages and so on. Moreover, most studies utilize heart rate segmentations generated by the ECG equipment, which still need patients wear electrodes to record original signals.

This study aims to develop an automatic sleep apnea syndrome severity assessment system, which is non-invasive, reliable, and can be easy-deployed in home environment, to help patients or their care-givers recognize the severity of sleep apnea syndrome timely and save medical treatment time. In this paper, we employ an unobtrusive sleep monitoring system (RS-611, http://www.risingsuntec.cn/04-product/product.ht) to record the original BCG signal in home environment, which is a certificated medical device and has been successfully applied in the detection of both heart rate variation information and breathing-related information [26]. By exploring the BCG signal, we propose an automatic sleep-related breathing event detection algorithm named Physio_ICSS which is based on the iterative cumulative sums of squares (ICSS) and can overcome the event segmentation issue effectively. Moreover, by integrating ICSS with the practical factors of sleep-related breathing events, Physio_ICSS is much more adaptive and time-efficient. Finally, the sleep apnea syndrome severity is obtained by calculating the AHI. To the best of our knowledge, this study is the first attempt to segment BCG signals for the detection of sleep-related breathing events and assess the sleep apnea syndrome severity according to the segmentation results. To sum up, the contributions of this paper mainly lie in three aspects:

First, we propose a framework for sleep apnea syndrome severity assessment by using only the BCG signal, which is obtained in an unobtrusive way. Compared with other physiological signals, such as ECG and SpO_2_, the most significant advantage of BCG is that users don’t have to attach any surface electrodes on their bodies during the recording process. However, there are also several disadvantages that limit the construction of an effective sleep apnea syndrome measurement model, such as poor data quality, low reliability, and high noise. To this end, we put forward a fine-grained signal preprocessing framework, including wavelet analysis as well as RR interval extraction and correction. Meanwhile, we also make full use of the rich content contained in the BCG signal, i.e., both the heart rate variation and breathing-related information.

Second, existing studies mainly adopt the equal-length segmentation scheme when detecting sleep-related breathing events. In other words, the original signals are first divided into equal-length fragments (e.g., 1min, 30s and 10s), and then the detection algorithm will be performed on the fragments. The main problem is that the number of sleep-related breathing events is not necessarily the same as the number of segments, as one segment might contain multiple sleep-related breathing events and a long sleep-related breathing event could appear in multiple segments. While there have been studies that utilized unequal-length segmentation scheme, they have not fully considered the practical factors of sleep-related breathing events. Moreover, existing studies usually explore multiple signals to achieve a better detection performance, which undoubtedly leads to heavy computation cost. To address the above challenges, we propose a sleep-related breathing event detection framework based on unequal-length segmentation, in which several practical factors have been taken into account to improve the performance, including the lasting duration limitation and the possible occurrence sleep stages of sleep-related breathing events.

Finally, by exploring the RR interval fragments, we extract a set of features from three different domains to characterize sleep-related breathing events. In particular, by fusing 8 time domain features, 8 frequency domain features and 2 nonlinear domain features, we build an efficient classification model which can identify sleep-related breathing events with the accuracy 97.57%.

The rest of this paper is organized as follows. In Section II, we introduce the data acquisition procedure, including the description of the sleep monitoring system and the summary of the experimental data. Section III provides the details of our proposed automatic sleep apnea syndrome severity assessment framework, which consists of 3 main steps, i.e., data preprocessing, sleep-related breathing event detection and severity evaluation. Based on a real-world dataset of 136 individuals, Section IV reports the experimental evaluation results of the proposed method. In Section V, we compare our results with existing studies, and summarize the contributions and shortcomings of our work. Finally, Section VI concludes the paper.

## Materials and methods

### Experimental data acquisition

**Fig 1:**
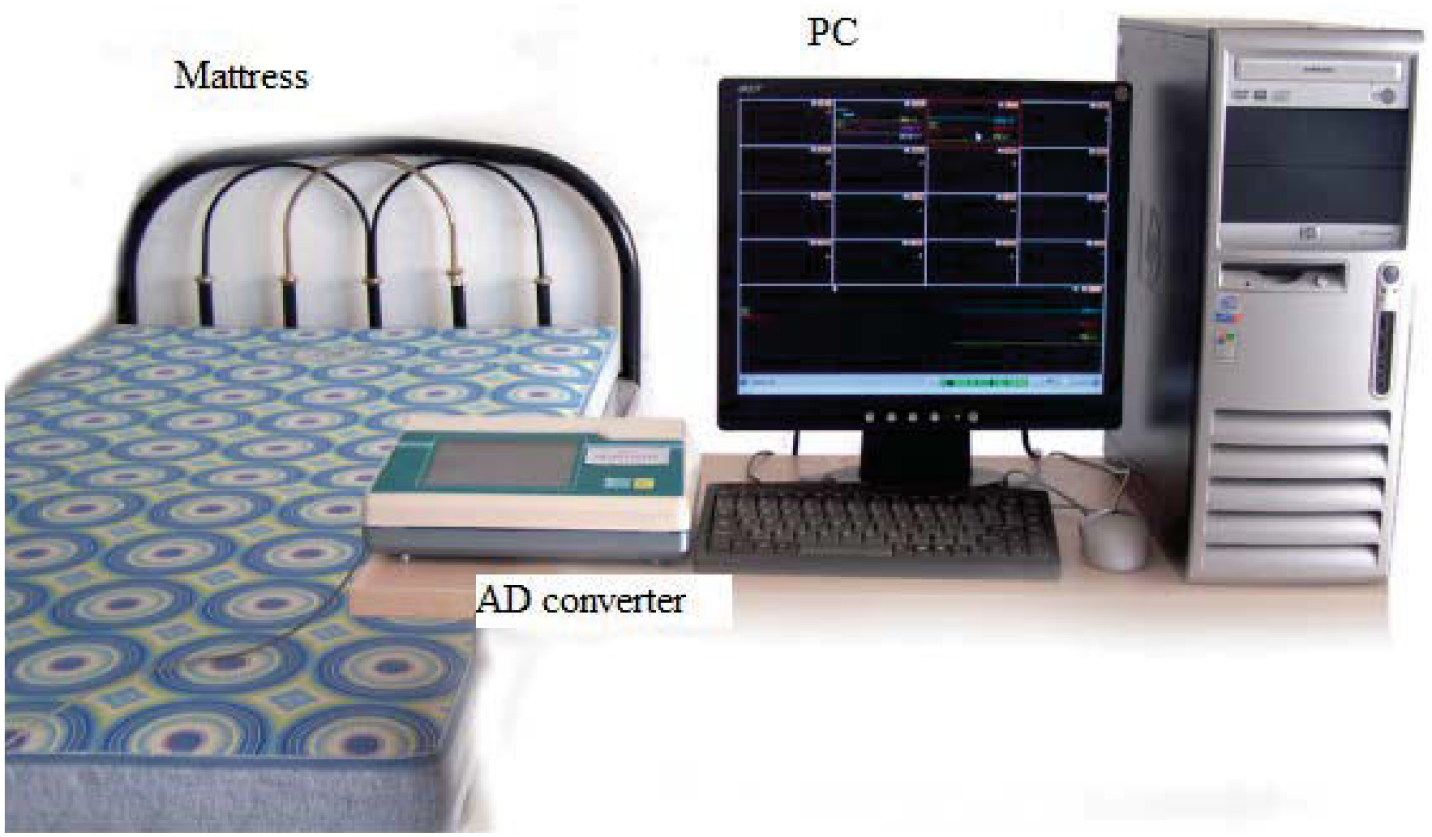
The sleep monitoring system.

In this work, in order to acquire the original BCG signal in home environment, we develop an unobtrusive sleep monitoring system, which not only reflects the heart rate variation information but also the breathing-related information. The system consists of a micro-movement sensitive mattress (MSM), an analog-digital (AD) converter and a terminal PC, as shown in Fig 1.

In Fig 1, MSM is the main functional part of the system, which includes four sectors and each sector is embedded with a set of pressure sensors to sense the movements of different body parts. In fact, sensors in different sectors can also check with each other to reduce measurement noise. Such a non-intrusive system would introduce less sleep disturbance and has the ability to monitor users over multiple nights.

**Fig 2:**
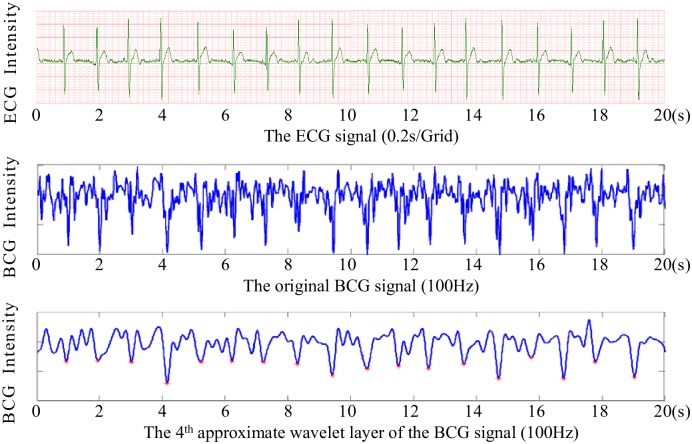
Performance of the micro-movement sensitive mattress.

When a subject lies on the mattress, pressure changes caused by heartbeat, breathing and other body movements will be captured by the embedded pressure sensors, and further converted to digital signals by the 16-bit resolution AD converter, forming a time series of composite pressure data. Afterwards, drift compensation and digital filtering is performed to separate BCG signal from the original composite pressure signals. In particular, to demonstrate the performance of the micro-movement sensitive mattress, we present a slice (20 seconds) of the obtained BCG signal (sub-figure in the middle) as well as the corresponding ECG signal (sub-figure on the top) recorded with Prince 180D (http://www.healforce.com/en/), as shown in Fig 2. We find that the original BCG signal can fully reflect the heart rate information, where each wave trough of the BCG time series corresponds to a local minimum point of the ECG time series. Furthermore, as there are still much high-frequency noise in the waveform, we need to develop an effective algorithm to extract RR intervals (i.e., identify a set of local minimum points) from the original BCG time series. For example, we can adopt multi-resolution wavelet analysis to parse the original BCG signal, and then identify local minimum points based on the approximate wavelet layer (sub-figure on the bottom). More details about how we can obtain RR intervals based on the BCG signal will be presented in the data preprocessing section.

In the present work, 136 subjects who took one night’s sleep with the monitoring system were studied, and all of them had provided verbal consent to participate in the study. The reason why written consent had not been obtained is that only those who agreed to share their data for scientific research were invited to participate the experiment. Meanwhile, all the data were analyzed anonymously. The Medical Experimental Ethical Inspection Institute of Northwestern Polytechnical University had approved our study as well as the above consent procedure (No. 20160173).

Among these 136 participants, there were 41 healthy subjects (26 males, 15 females) as the control group, 23 mild sleep apnea syndrome suffers (14 males, 9 females), 34 moderate sleep apnea syndrome suffers (20 males, 14 females) and 38 severe sleep apnea syndrome suffers (24 males, 14 females). The sleep time durations of all recordings were no less than 6 hours, and nearly 9 hours at most. Subjects suspected of having any cardiovascular or lung diseases were excluded in this research. For comparison purposes, we also captured the ECG, SpO_2_ and respiration signals simultaneously by utilizing the conventional PSG equipment. In addition, the ground truth of heart beat intervals, sleep apnea and the hypopnoea events were manually identified by sleep experts according to the manual for scoring of sleep and associated events [27]. The mean age, body mass index (BMI), and AHI are summarized in Table 1.

**Table 1:** Statistics of the experimental data set.

	All	Healthy	Mild	Moderate	Severe
**Number**	136	41	23	34	38
**Ages**	53.1 ± 5.1	47.8 ± 4.2	54.0 ± 4.1	54.6 ± 3.3	56.9 ± 3.0
**BMI**	26.9 ± 4.0	22.9 ± 2.2	24.8 ± 2.4	28.9 ± 2.4	30.8 ± 2.0
**AHI**	19.6 ± 15.4	2.4 ± 1.3	11.1 ± 4.4	23.9 ± 2.8	39.5 ± 7.1

Specifically, for each measure reported in Table 1, we performed *t-test* to detect whether there are significant differences among groups (i.e., healthy, mild, moderate and severe). In case of the age measure, as shown in Table 2, we observe that there do exist certain difference, which is consistent with the findings of existing studies [1–3]. However, even though the occurrence rate of sleep apnea syndrome increases gradually with the age, such a correlation is very slight and we should not take the user’s age as an important feature when assessing sleep apnea syndrome (e.g., it is difficult to differentiate mild suffers from moderate ones). The *t-test* result of the body mass index is shown in Table 3. Accordingly, while all the *p-values* among healthy, mild and moderate groups are larger than 0.05 (i.e, indicating there is no significant difference), the *p-values* between the severe group and the other three groups are much smaller (i.e., indicating there is significant difference). Therefore, we can conclude that subjects with severe obesity are more likely to develop severe sleep apnea syndrome. In case of the apnea hypopnoea index, according to Table 4, we find that there are significant differences among all the four groups (*p-values* ranging from 10^−17^ to 10^−54^), which is in accordance with the definition of sleep apnea syndrome by the American Academy of Sleep Medicine [8].

**Table 2:** *p-values* of the age among different groups.

	Healthy	Mild	Moderate	Severe
**Healthy**	/	<10^−7^	<10^−11^	<10^−17^
**Mild**	<10^−7^	/	0.504	0.002
**Moderate**	<10^−11^	0.504	/	0.003
**Severe**	<10^−17^	0.002	0.003	/

**Table 3:** *p-values* of the body mass index among different groups.

	Healthy	Mild	Moderate	Severe
**Healthy**	/	0.0018	<10^−17^	<10^−27^
**Mild**	0.0018	/	<10^−8^	<10^−15^
**Moderate**	<10^−17^	<10^−8^	/	<10^−4^
**Severe**	<10^−27^	<10^−15^	<10^−4^	/

**Table 4:** *p-values* of the apnea hypopnoea index among different groups.

	Healthy	Mild	Moderate	Severe
**Healthy**	/	<10^−17^	<10^−54^	<10^−47^
**Mild**	<10^−17^	/	<10^−19^	<10^−25^
**Moderate**	<10^−54^	<10^−19^	/	<10^−18^
**Severe**	<10^−47^	<10^−25^	<10^−18^	/

Additionally, for each of the above three measures, we also performed *t-test* on gender of the participants. Results show that while there are no significant differences in ages among males and females (*p-value* = 0.637), male participants tend to have higher body mass indexes (the average body mass indexes of males and females are 27.5 and 26.0, and the *p-value* is 0.029) as well as higher apnea hypopnoea indexes (the average values are 22.0 and 15.8, and the *p-values* is 0.024), indicating that males are more likely to develop sleep apnea syndromes.

### Sleep apnea syndrome severity assessment framework

**Fig 3:**
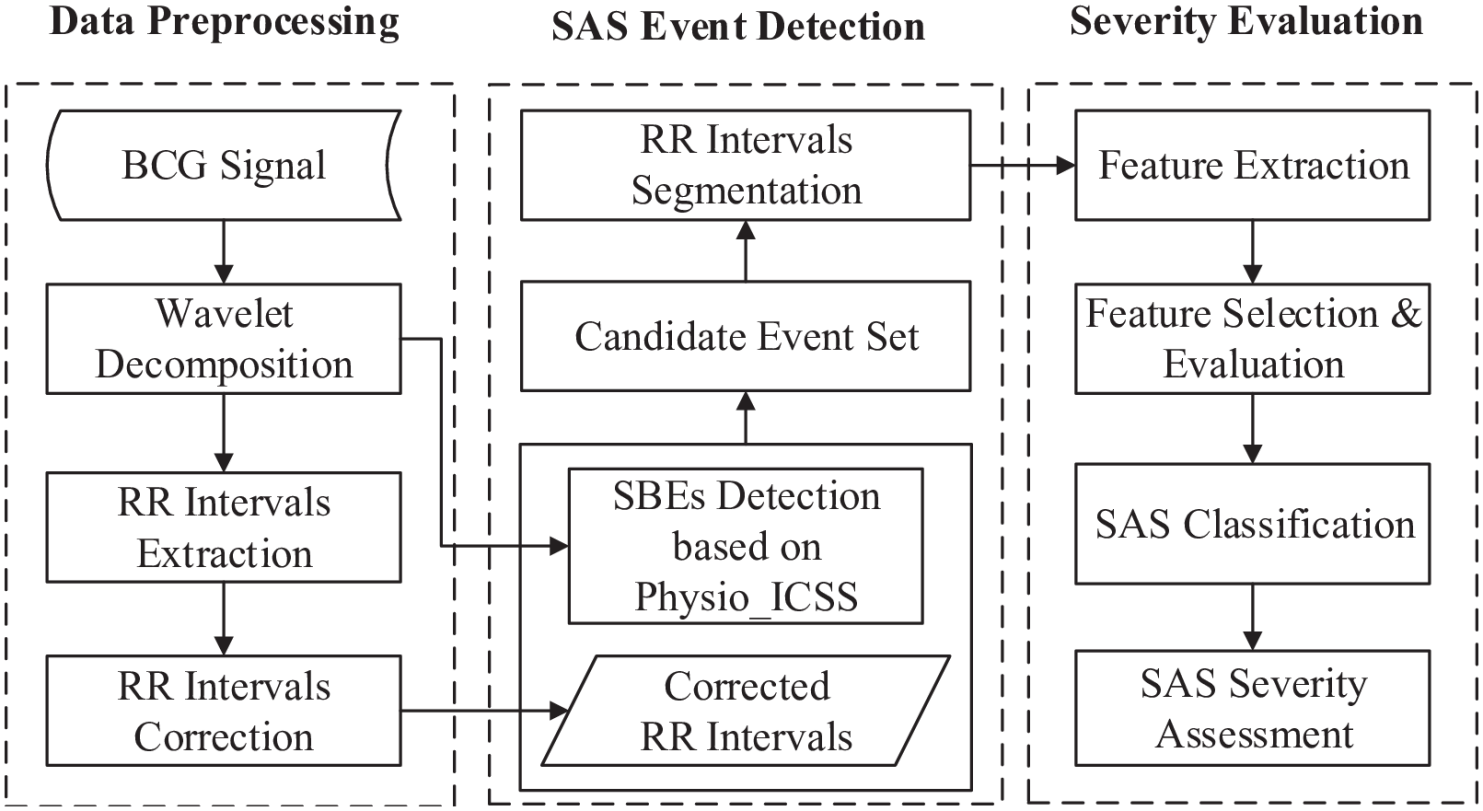
Framework of the proposed approach.

In this paper, we aim to automatically assess the severity level of sleep apnea syndrome by exploring the BCG signal. The proposed approach can be divided into three stages, namely data preprocessing, sleep-related breathing event detection and severity evaluation, as shown in Fig 3.

During the data preprocessing stage, we apply the multi-resolution wavelet analysis to refine the time-frequency information of the original BCG signal. Afterwards, beat-to-beat (RR) heart rate intervals of the BCG signal are obtained and corrected.

In the second stage, sleep-related breathing events are detected based on the proposed Physio_ICSS algorithm, which is much more effective in capturing small variation changes in the BCG signal, compared with the original ICSS algorithm. Specifically, the small variation changes in BCG signal are mostly caused by sleep-related breathing events in consideration of their amplitude characteristics [28], which can be deemed as the breathing-related information of BCG signals. To effectively identify events from the candidate event set, we analyze the heart rate information in the BCG signal, and segment the corrected RR intervals according to the output of the Physio_ICSS algorithm.

In the final stage, 8 time domain features, 8 frequency domain features and 2 nonlinear features are extracted from the obtained RR intervals. Moreover, we train and employ three classification models in our experiments, i.e., the *k*-Nearest Neighbor classifier, the random forest classifier and the SVM classifier. Based on the classification results, the severity of sleep apnea syndrome can be evaluated by calculating the AHI.

#### Data preprocessing

In this section, we will present how to preprocess the original BCG signal and how to extract the RR intervals of the BCG signal.

**BCG signal preprocessing**. The original BCG signal describes the periodical chest vibration, which is mainly modulated by the movement of the heart beat and respiration. BCG provides a non-invasive way to evaluate the user’s heart condition. When cardiac dysfunction occurs, there will be certain anomalies in the corresponding BCG signal. As shown in Fig 3, we can clearly find out that although the original BCG signal can fully reflect the heart rate information, there are still much redundant high-frequency noise in the waveform, which has a negative effect on the extraction of RR intervals.

The wavelet analysis focuses on localize analysis in both time domain and frequency domain, which can be used to analyze signals at different scales and resolutions, especially for non-stationary physiological signals [29, 30]. In practical applications, we can use different wavelet-based functions for different kinds of signals. Specifically, considering that different wavelet analysis resolutions contain different information, we adopt the multi-resolution wavelet analysis to parse the original BCG signal.

**Fig 4:**
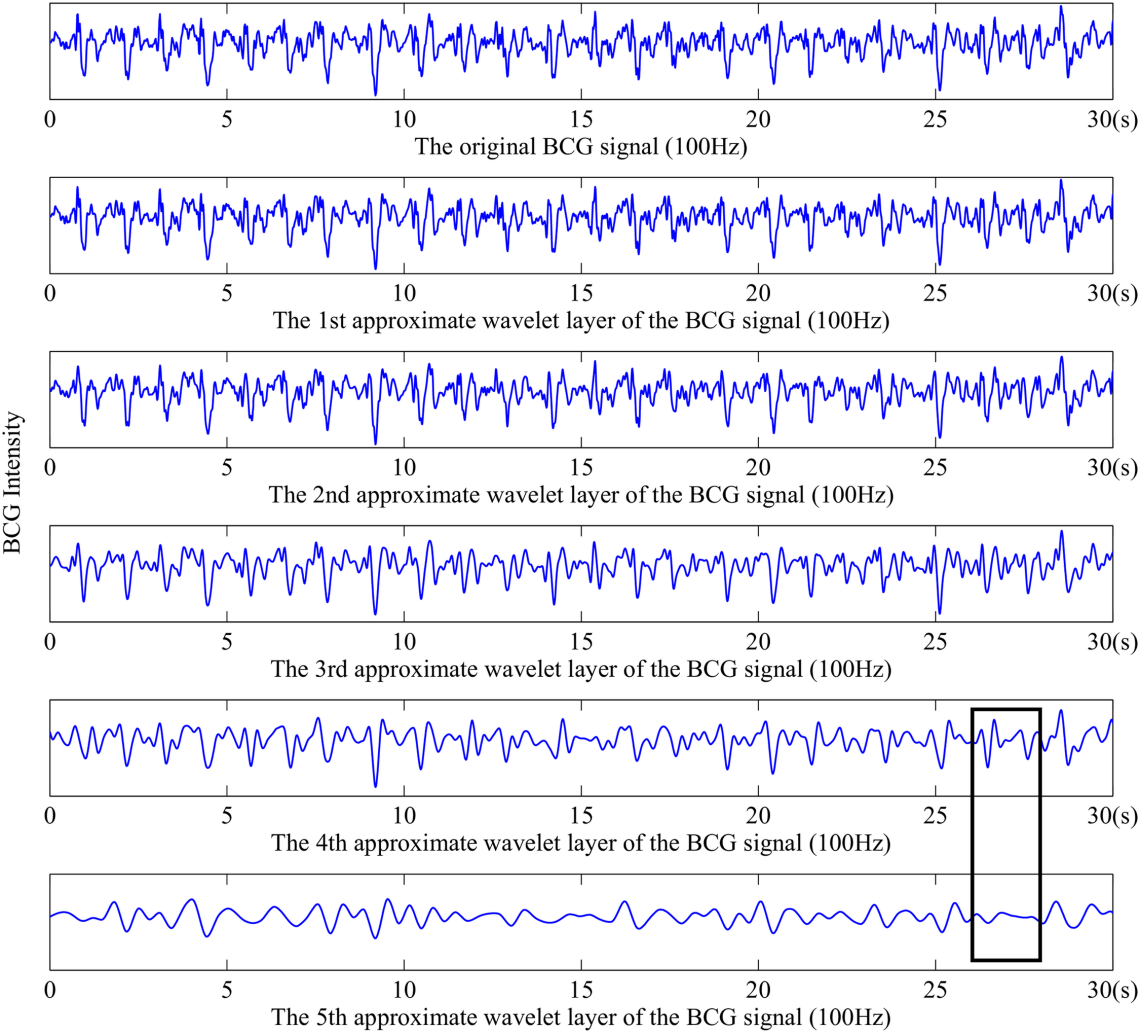
Multi-resolution wavelet analysis results of the original BCG.

Fig 4 demonstrates the multi-resolution wavelet analysis results of the original BCG with the Symlets 8 wavelet-based function. From top to bottom, the redundant high-frequency glitches decrease gradually, and the wave profile becomes clear. While the 5th layer is smoother than any other layers, the parts in the black rectangles reveal that it contains waveform distortion at a certain degree. Thereby, we choose the fourth approximation layer to extract RR intervals.

**RR intervals extraction and correction**. RR intervals measure the duration of heart beat cycles, and the RR interval time series is the foundation of calculating different evaluation indexes of heart rate variability. In this work, we take second (s) as the unit of time to evaluate RR intervals. According to Fig 4, we can see that there are still noise around the wave peaks in the fourth approximation wavelet layer. Therefore, we choose to obtain RR intervals by calculating the time span between adjacent wave troughs. Specifically, an overlapped sliding window method was applied to detect all the wave troughs, and the minimum point of each window will be regarded as candidate times of heart beats. According to experimental results, the window size was set as 100 samples (i.e., 1 second) and the sliding step was set as 80 samples. In particular, the reason why an overlapped sliding window method was adopted is that, no matter we choose a larger or smaller window size, there always exist some RR intervals longer or shorter than the length of a window. If a non-overlapped sliding window method was adopted, it will lead to quite a number of leak checked wave troughs as well as fault checked ones, which result in either too long or too short RR intervals. For instance, suppose that a user’s average heart rate is 60 beats per minute, the length of 100 samples should be a suitable window size. In case that the user suffers from sleep apnea syndrome, she/he is very likely to have some longer RR intervals (e.g., 1.1 second) as well as some shorter ones (e.g., 0.9 second), which will severely decrease the performance of a non-overlapped sliding window method less efficient.

With the overlapped sliding window method, the vast majority of wave troughs can be detected correctly, as shown in Fig 2. However, even though the overlapped sliding window method overcomes some shortcomings of the non-overlapped method, there are still some leak checks and fault checks of RR intervals, which might due to the movements of other body parts.

**Fig 5:**
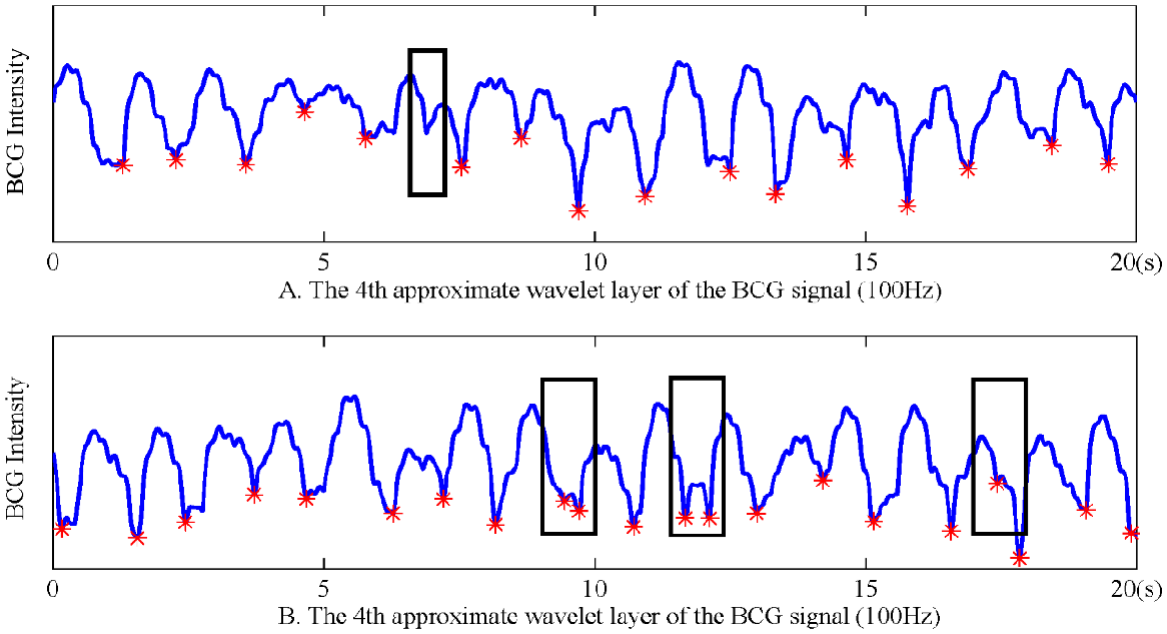
An example of leak and fault checked RR intervals. A: Leak check. B: Fault check.

Examples of leak checked and fault checked RR intervals are given in Fig 5A and 5B, respectively, where the parts in black rectangles correspond to the leak check and fault check of RR intervals.

To address the challenge of leak check and fault check of RR intervals, we propose a novel RR correction algorithm (as shown in Algorithm 1), which is based on the fact that most of the RR intervals have been correctly detected with the sliding window method.

**Algorithm 1**. The RR Correction Algorithm

**Require:**

- *RR*, the original *RR* interval time series
- 2*ω*, the window length

**Ensure:**

- *RR*_*corrected*_, the final RR interval time series

1: *n* ← length(*RR*);

2: **for** each *i* ∈ [1, *n*] **do**

3:  $\bar{RR_{i}}\leftarrow$ the mean length of 2*ω* intervals near *RR*_*i*_

4:  **if** $RR_{i}>1.5*\bar{RR_{i}}$ **then**

5:   *k* ← $\left\lfloor \frac{RR_{i}}{\bar{RR_{i}}}+\frac{1}{2}\right\rfloor$ (the number of potential *RR*)

6:   *RR*_*inew*_ ← *RR*_*i*_/*k*

7:   *RR*_*i*_ ← *RR*_*inew*_, *inew* = 1, 2, 3, …, *k*

8:  **end if**

9:  **if** $RR_{i}<0.5*\bar{RR_{i}}$ **then**

10:   *maxNeighbour* ← max{*RR*_*i*−1_, *RR*_*i*+1_}

11:   *minNeighbour* ← min{*RR*_*i*−1_, *RR*_*i*+1_}

12:   **if** $maxNeighbour-minNeighbour<\bar{RR_{i}}$ **then**

13:    *maxNeighbour* ← *maxNeighbour* + 0.5 ∗ *RR*_*i*_

14:    *minNeighbour* ← *minNeighbour* + 0.5 ∗ *RR*_*i*_

15:   **else**

16:    *minNeighbour* ← *minNeighbour* + *RR*_*i*_

17:   **end if**

18:  **end if**

19: **end for**

To extract and correct RR intervals, we first apply the fixed-size sliding window method to obtain the preliminary result, and then refine it based on the proposed RR correction algorithm. Specifically, for each candidate interval *RR*_*i*_ in the original *RR* interval time series, the average length of its 2*ω* neighbor intervals is defined as $\bar{RR_{i}}$ (line 3). In case that the length of *RR*_*i*_ is longer than 1.5*$\bar{RR_{i}}$, we define that a leak check happened and the RR interval time series should be refined. In particular, we first calculate the number of RR intervals that should be inserted (line 5), and then replace *RR*_*i*_ with the new intervals (lines 6-7). In case that the length of *RR*_*i*_ is shorter than 0.5*$\bar{RR_{i}}$, we define that a fault check happened and *RR*_*i*_ should be eliminated. Particularly, if $maxNeighbour-minNeighbour<\bar{RR_{i}}$, *RR*_*i*_ will be divided equally by its two neighbors (lines 13-14). Otherwise, *RR*_*i*_ will be merged to the closer neighbor (line 16).

**Fig 6:**
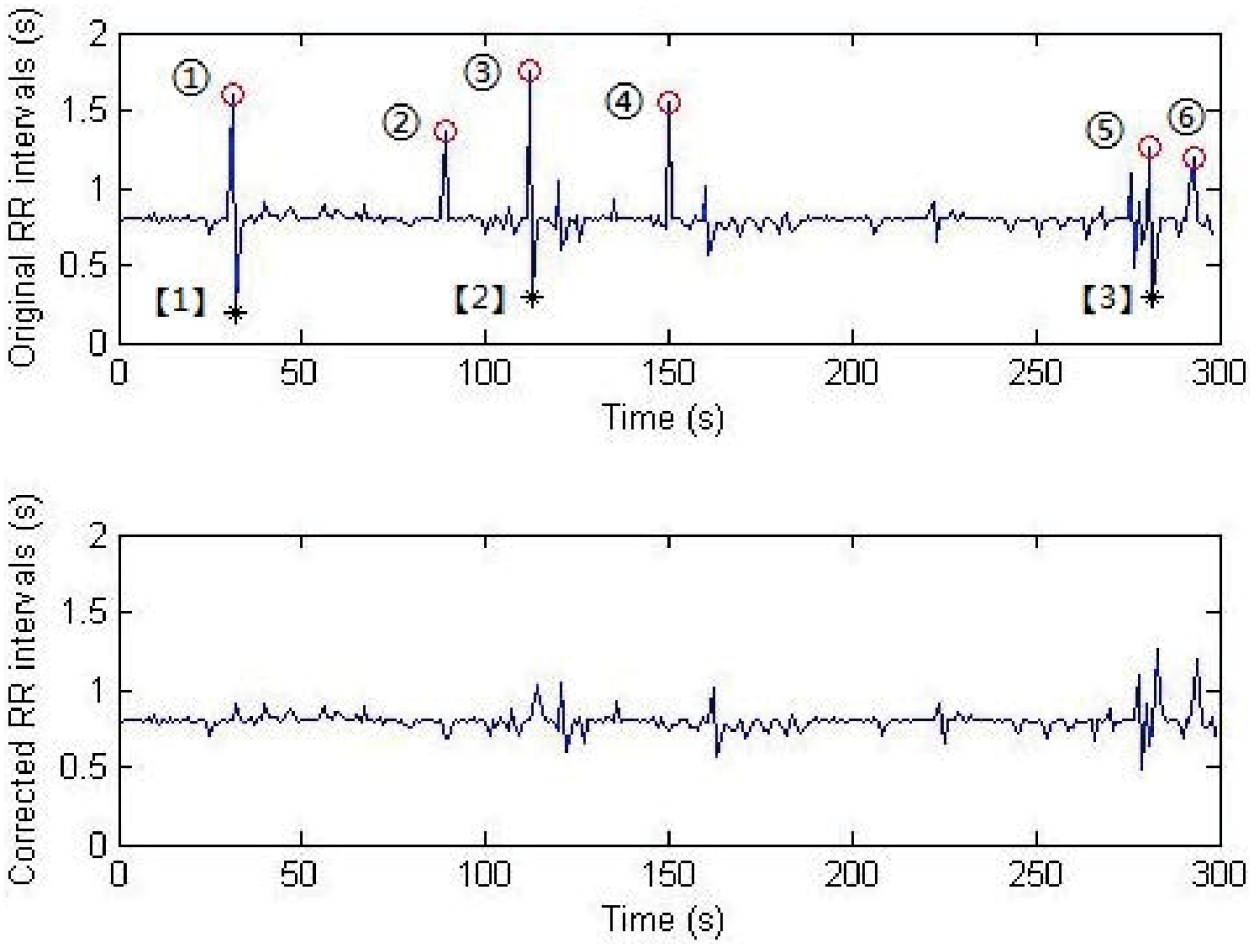
Performance of the proposed RR correction algorithm.

An example to demonstrate the difference of RR sequence before and after correction is shown in Fig 6. We can find that there are 6 suspected leak checked RR intervals (i.e., ①-⑥) and 3 suspected fault checked RR intervals (i.e., [1]-[3]) in the original RR sequence. On one hand, the correction algorithm will deal with the leak checked RR intervals by splitting them with the average length of the nearby RRs (lines 5-7). On the other hand, different methods will be used to handle the fault checked intervals according to the comparison result of two adjacent RRs (lines 12-17). According to Fig 6, we can find that after correction, there is hardly any fault checks in the RR interval time series. It should be pointed out that ⑤ and ⑥ are not real leak checks, as they do not satisfy the definition of leak checks.

Moreover, to evaluate the performance of the proposed RR interval extraction and correction algorithms, experiments were designed based on synthetic data as follows. We first generated a sine wave series of 10,000 periods and each period includes 100 samples, resulting in a synthetic time series with 1,000,000 samples. Secondly, white Gaussian noise was added to the synthetic signal, by setting the signal-noise ratio as 10. Afterwards, 1,000 data points, whose *x*-axis ranged from 1 to 1,000,000 and *y*-axis ranged from -0.8 to -1.2, were randomly generated to replace the corresponding data points in the synthetic signal, i.e., 10% potential leak and fault checks were introduced. By setting the size of sliding window as 100 samples and the sliding step as 80 samples, we repeated the experiments for 100 times. While the average error rate (i.e., the ratio of leak and fault checks) of the RR interval extraction algorithm was 3.26%, it declined to 1.48% after correction, which validates the effectiveness of the RR correction process.

In addition, we also compared the BCG-based RR interval series with the one derived from ECG. Specifically, we simultaneously collected the ECG and BCG data of 10 subjects for one night, and the average error rate was around 0.25 to 1.08 beats per minute, indicating that we can obtain satisfactory RR intervals based on the BCG signal. An example was shown in Fig 2, where a slice of 20-second ECG signal (sub-figure on the top) and the corresponding BCG-based RR intervals (sub-figure on the bottom) were presented.

Furthermore, considering the non-uniform sampling property of the RR interval time series, which might affect the result of nonlinear analysis, we re-sample the corrected RR series using cubic spline interpolation with a sampling rate of 4 Hz.

#### Detection of sleep-related breathing events

In this section, we will elaborate the proposed Physio_ICSS algorithm and present how to segment the time series of RR intervals using the algorithm.

**Physio_ICSS algorithm**. As above mentioned, the original BCG signal includes both heart rate information and breathing-related information. When sleep-related breathing events occur, the user’s breathing amplitude will significantly decrease, which will lead to the drop of the BCG amplitude as well. In fact, the small variations in amplitude caused by sleep-related breathing events can be regarded as structural changes of the BCG signal. Based on the concept of iterative cumulative sum of squares, we propose a sleep-related breathing event detection algorithm named Physio_ICSS (S1 Algorithm), which is able to detect sudden structural changes in long time series [31, 32]. For more details, please refer to the Supporting Information.

Compared with the original ICSS algorithm, on one hand, the proposed Physio_ICSS algorithm has less strict constraints to the data, which is much more suitable for the analysis of physiological signals. On the other hand, the algorithm also makes full use of the practical factors of sleep-related breathing events, i.e., the possible duration and occurring sleep stages of sleep-related breathing events. Therefore, with only BCG signal, the Physio_ICSS algorithm could overcome the three main shortcoming of existing approaches and detect sleep-related breathing events effectively. Moreover, as AHI is calculated at the hour scale, the Physio_ICSS algorithm only need be performed once per hour rather than the whole night. In other words, the algorithm will be repeated by 6 to 9 times each night, which significantly reduces the overall time overhand.

**Segmentation of RR intervals**. Based on the proposed Physio_ICSS algorithm, we can obtain the starting and ending points of all the detected fragments which are stored in the *PF* vector, and all the suspect sleep-related breathing events are contained in these fragments. Meanwhile, we also obtain a status array *S*, which indicates whether a certain fragment need be further examined, i.e., fragments shorter than 10s will be directly excluded which are impossible to be sleep-related breathing events. It is necessary to point out that not all of the detected fragments contain sleep-related breathing events, and some of them are NSBEs fragments (NSBEs are used to denote fragments that do not contain sleep-related breathing events for the convenience of description, which include segments between the ending point and the starting point of two adjacent sleep-related breathing events as well as those detected as sleep-related breathing events by mistake). Therefore, to calculate AHI, some further analysis is needed to identify all the suspect fragments of sleep-related breathing events automatically.

According to existing studies [33, 34], the effect of sleep apnea syndrome can be observed within cardiovascular system, and also might be accompanied with cyclic variations in RR intervals. Therefore, to further identify sleep-related breathing events from the detected fragments, we perform heart rate variability analysis for all the candidate fragments according to the status array. Moreover, as the Physio_ICSS algorithm is not absolutely perfect for the detection of sleep-related breathing events, some structural change points might be leak checked, leading to abnormal fragments with a long range. Therefore, the length of a segment is empirically defined as min{10mins, (*PF*_*i*+1_ − *PF*_*i*_)}.

#### Severity evaluation

In this section, we will present how to evaluate the severity of sleep apnea syndrome by identifying sleep-related breathing events.

**Feature extraction**. For each unequal-length RR segments that need be further examined according to the status array, a set of features would be extracted for the classification of SBEs and NSBEs. Specifically, we have defined 18 different features, including 8 time domain features, 8 frequency domain features and 2 nonlinear features, as shown in Table 5.

**Table 5:** List of extracted features.

Type	Features	Description
Time	*Mean*	the mean value of RR segments
	*Var*	the variance of RR segments
	*Max*	the maximum of RR segments
	*Min*	the minimum of RR segments
	*RMSSD*	the root mean square of adjacent RRs in the segments
	*SDSD*	the standard deviation of adjacent RR differences in the segments
	*PNN50*	the percentage of RR segments longer than 50s
	*CV*	the variation coefficient of RR segments
Frequency	*vLF*	the power in vLF band
	*LF*	the power in LF band
	*HF*	the power in HF band
	*vHF*	the power in vHF band
	*LF*_*nor*_	the normalized power in LF band
	*HF*_*nor*_	the normalized power in HF band
	*LF*/*HF*	the ratio of power in LF and HF band
	*TF*	the total power in the whole band
Nonlinear	DFA	the short-term coefficient of detrended fluctuation analysis
	SampEN	the sample entropy value with *r* = 0.2*STD

The 8 time domain features are the *mean*, the *variance*, the *maximum*, the *minimum*, the *RMSSD*, the *SDSD*, the *PNN50* and the *CV* of RR fragments, which can reflect the variations of RR segments over time in different angles.

However, these time domain features cannot effectively evaluate the balance of the autonomic nervous system when sleep apnea syndrome happens. Nevertheless, frequency domain features have been proved to be effective ones to assess variations of the autonomic nervous system. Specifically, in this paper we adopt the autoregressive model (AR model) to conduct frequency-domain analysis, which has smoother spectrum curve and higher resolution in different bands [35], compared with classic spectrum estimation methods that are based on the Fourier Transform theory. In particular, the order of the AR model is set as 16 in our work, which can clearly reveal the center frequency of the HF and LF bands [36]. The frequency features we used are the *vLF* (0.0033Hz-0.04Hz) power, the *LF* (0.04Hz-0.15Hz) power, the *HF* (0.15Hz-0.4Hz) power, the *vHF* (0.4Hz-0.5Hz) power, the *TF* (0Hz-0.5Hz) power, and the ratio of the power in LF and HF band *LF*/*HF*. Moreover, in consideration of the inter-subject variability, we introduced the normalized *LF* and *HF* (i.e., *LF*_*nor*_ and *HF*_*nor*_) as follows:

$$\begin{array}{c} LF_{nor}=LF/\left(LF+HF\right), \end{array}$$ (1)

and

$$\begin{array}{c} HF_{nor}=HF/\left(HF+LF\right). \end{array}$$ (2)

Meanwhile, we also extracted nonlinear features to measure the variability and complexity of a fragment, based on the nonlinear methods Detrended Fluctuation Analysis (*DFA*) [37] and Sample Entropy (*SampEN*) [38, 39]. Specifically, we used the short-term coefficient of *DFA* to evaluate the inner correlation of RR time series, and set the parameter *s* (i.e., the length of a fragment) as 40 to 320 based on the frequency of heartbeat. Similarly, there are also two important parameters in the *SampEN* algorithm, i.e., the vector detail level *m* and the threshold *r*, which are set as *m* = 2 and *r* = 0.2*STD (standard deviation) respectively, according to existing studies [37].

**Classification of SBEs and NSBEs**. In order to identify sleep-related breathing events and classify the segments into two classes, i.e., NSBEs and SBEs, we mainly adopted three different types of classification models in the present work, including the *k*-Nearest Neighbors (kNN), the Random Forest (RF) and the Support Vector Machine (SVM).

Specially, the kNN classifier is a non-parametric method used for classification and regression. It is a type of instance-based learning algorithm, where the function is only approximated locally and all computation is deferred until classification. A shortcoming of the kNN algorithm is that it is sensitive to the local structure of the data.

The RF classifier is composed of a set of discrete decision tree models, which can overcome the disadvantages that a simple decision tree brings during the classification procedure, such as over-fitting and local rather than global optimal solution issues. For a data sample, the RF classifier determines its class by counting the voted numbers given by different decision trees, and the class with maximum voted numbers will be the final class. Actually, the RF classifier has advantages in evaluating attribute relationship and handling large dataset, and it can also effectively handle data noise and outliers.

SVM is a simple and effective two-class classifier. Based on the training data, SVM can construct an optimal hyperplane by maximizing the margin between the two classes, and then use it to separate the testing data. Moreover, as for some non-linear classification problems, SVM can map the data into a high dimensional space by using different kernel functions, and then solve them as linear classification problems.

To eliminate the redundant features as well as validate the performance of different feature sets, we adopted three feature selection strategies which are *Null* (i.e., using all the extracted features), *Information Gain* based feature selection and *Sequential Forward* feature selection. Specifically, in case of information gain based feature selection, we kept all the features whose information gain were larger than 0. In case of sequential forward feature selection, for each given set of parameters, features were sequentially added to the candidate set until the addition of further features does not decrease the misclassification rate.

Moreover, for each candidate set of parameters, we adopted unconstrained nonlinear optimization to perform fine-grained parameter tuning, aiming at achieving optimal performance. Meanwhile, a nested cross validation procedure was conducted to obtain reliable and unbiased classifiers. Specifically, we chose 10-fold cross validation for the outer loop and 5-fold cross validation for the inner loop.

## Experimental results

### Experimental setup

As aforementioned, we only considered the REM and NREM sleep stages in the experiments, and the Wake sleep stage was excluded. In addition, to simplify the calculation of AHI and reduce the total time cost for the preliminary detection of sleep-related breathing events, we segmented the whole night’s sleep time (6h-9h) into multiple one-hour fragments (except the Wake sleep stage), and the fragment whose duration was less than one hour had been discarded. Furthermore, to obtain the ground truth of SBEs and NSBEs, a professional sleep physician was recruited to annotate the PSG data of 136 participants of the experiment, and a total number of 17,946 fragments were labeled as SBEs.

### Detection performance of sleep-related breathing events

**Fig 7:**
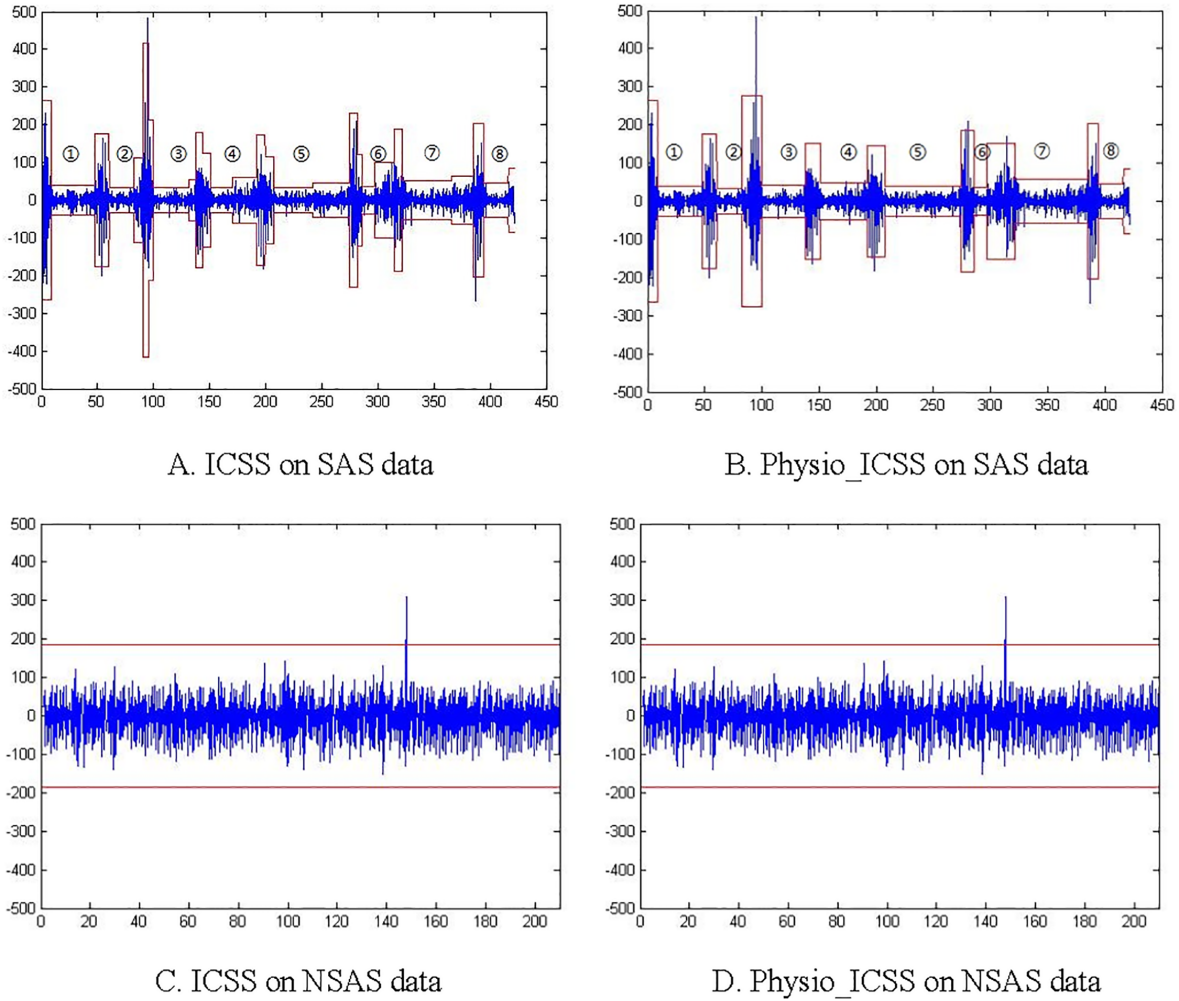
Sudden change detection performance of original ICSS and Physio_ICSS. A: ICSS on SAS data. B: Physio_ICSS on SAS data. C: ICSS on NSAS data. D: Physio_ICSS on NSAS data.

Both the original ICSS algorithm and the proposed Physio_ICSS algorithm are evaluated, and their performance on a piece of BCG data with 8 sleep-related breathing events are shown in Fig 7A and 7B. Meanwhile, Fig 7C and 7D present the results of the two algorithms on a piece of normal sleep data (i.e., NSAS). Specifically, in Fig 7, the horizontal axis stands for the time (seconds), and the vertical axis represents the relative amplitude. The threshold G* we used is 1.628, and the corresponding confidence level is 0.99, which are empirically determined based on experiments.

According to Fig 7A and 7B, we can find that the original ICSS algorithm detected more redundant structural changes (i.e., trivial changes in ③-⑦) than Physio_ICSS. In other words, if we directly utilize the original ICSS rather than Physio_ICSS, more sleep-related breathing events might be detected than the actual amounts, leading to the wrong calculation of AHI. Specifically, the main reason why Physio_ICSS performs better than original ICSS is that it takes the practical factors of sleep-related breathing events into the design of the algorithm, including the duration limitation and the possible occurrence sleep stages of sleep-related breathing events. In addition, Physio_ICSS is more suitable to unstable physiological signals (e.g., BCG) than the original ICSS, which used an improved testing statistics (i.e., *κ*_2_ = sup_*t*_|*T*^−1/2^ *G*_*t*_|).

Table 6 gives a comparison of the time cost between the original ICSS and Physio_ICSS. As the detection time is strongly related to the number of sleep-related breathing events, we only list the results of two different pieces of data as an example.

**Table 6:** Time consumption of the original ICSS and Physio_ICSS.

	ICSS	Physio_ICSS
**6-min data (AHI = 13)**	1.0397s	0.4162s
**60-min data (AHI = 48)**	224.7339s	21.4574s

Obviously, for one-hour data from a severe sleep apnea syndrome suffer, Physio_ICSS spends less than 1/10 time of the original ICSS, which can be regarded as a proof that it is reasonable to segment the whole night’s data into one-hour fragments. In addition, as for a shorter piece of data, Physio_ICSS still has some advantages over the original ICSS. In fact, the lower time consumption of Physio_ICSS is mainly due to the consideration of practical factors of sleep-related breathing events, which optimize the solution space and add some pruning limitations. Specifically, the experiment is conducted with Matlab on an Intel Core i5 PC with 4GB RAM running Windows 8.

According to the above discussion, we can find that the performance of Physio_ICSS is better than the original ICSS in both detection accuracy and time cost. Moreover, in order to evaluate Physio_ICSS comprehensively, we further introduce three metrics, namely, the error rate (*ER*), the sensitivity rate (*SR*) and the positive prediction rate (*PPR*), which are formally defined as follows:

$$\begin{array}{c} \left\{ \begin{array}{ccc} ER=(FN+FP)/Total\_Event\_Num & & \\ SR=TP/Total\_Event\_Num & & ,\\ PPR=TP/Total\_Detected\_Num & \end{array}\right. \end{array}$$ (3)

where True Positive (*TP*) refers to the number of SBEs that has been correctly identified, False Positive (*FP*) is the number of NSBEs that has been incorrectly identified as SBEs (fault checked), and False Negative (*FN*) represents the number of SBEs that has not been correctly identified (i.e., leak checked). Meanwhile, *Total_Event_Num* is equal to the sum of *TP* and *FN*, and *Total_Detected_Num* is defined as the sum of *TP* and *FP*. Obviously, a good sleep-related breathing event detection method should have low *ER*, and high *SR* and *PPR*.

**Fig 8:**
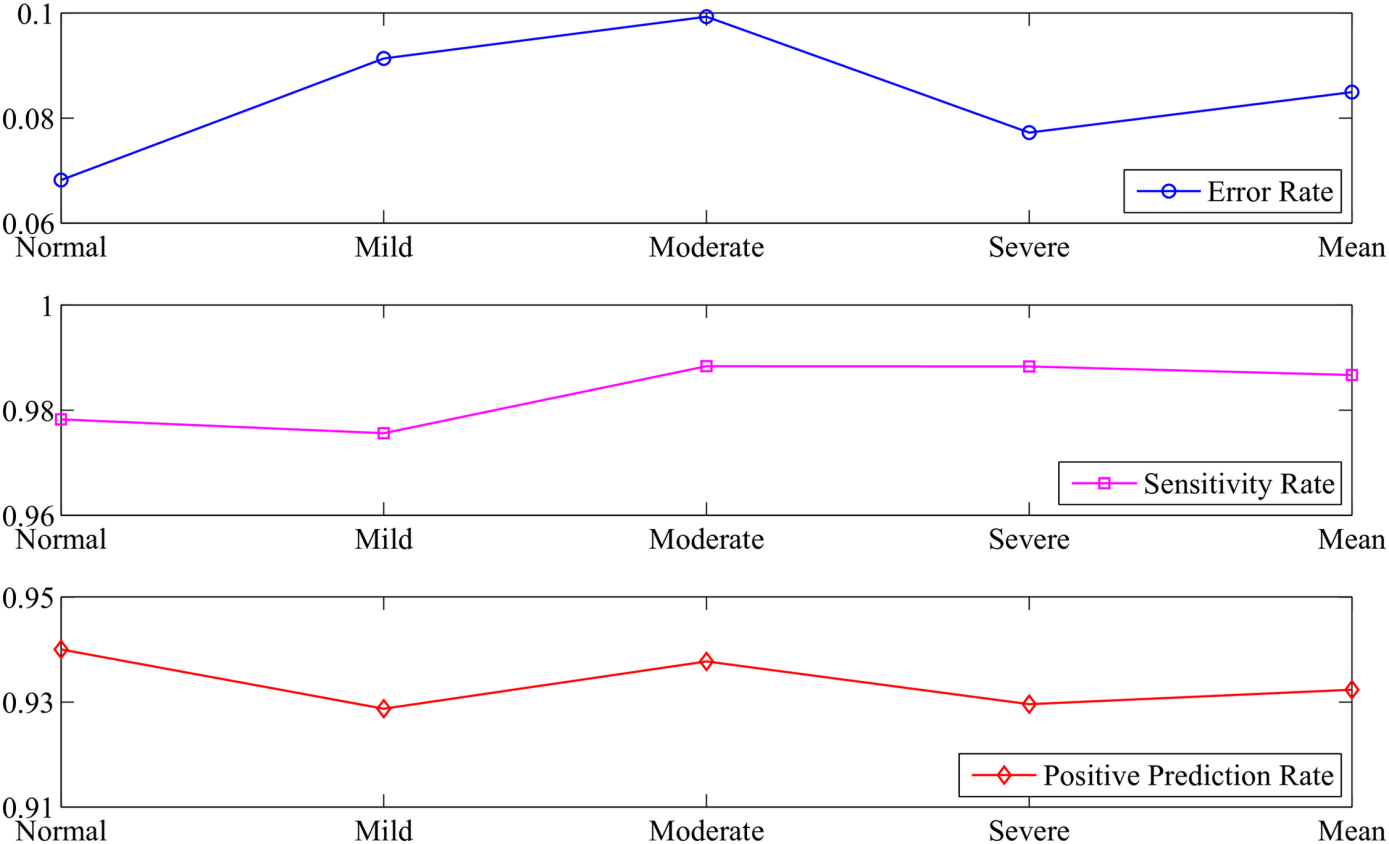
Performance of the Physio_ICSS algorithm.

Based on the proposed Physio_ICSS algorithm, a total number of 18,992 BCG fragments of 136 participants were identified as suspect SBEs. Compared with the ground truth obtained based on PSG, 1,285 NSBEs had been incorrectly identified as SBEs, and 239 SBEs had not been correctly identified. Specifically, the error rate, sensitivity rate and positive prediction rate for healthy subjects, mild sleep apnea syndrome sufferers, moderate sleep apnea syndrome sufferers, severe sleep apnea syndrome sufferers as well as all the participants were shown in Fig 8. Accordingly, we can see that Physio_ICSS has lower average *ER* (around 8.49%), higher mean *SR* (around 98.7%), and higher mean *PPR* (around 93.2%), indicating that the algorithm is able to detect sleep-related breathing events effectively.

### Severity evaluation performance

#### Performance of different classifiers

According to the above section, based on the proposed Physio_ICSS algorithm we detected most of the SBEs fragments (about 98.7%). However, there were still quite a number of NSBEs that had been incorrectly identified as SBEs (about 6.8%), which might impact the assessment of sleep apnea syndrome severity levels. Thereby, we need to further classify all the suspect SBEs by analyzing the corresponding RR interval time series by exploring the extracted features.

According to experimental results, there are two parameters that would significantly influence the performance of kNN, which are the number of nearest neighbors to find (NumNeighbors) and the distance metric (Distance). Therefore, we had mainly attempted different settings of these two parameters when optimizing the kNN classifier, while keeping other parameters just the same as their initial settings in Matlab. In particular, during experiments the number of nearest neighbors was set as {2^0^, 2^1^, …, 2^10^}, and the used distance metrics included ‘Cosine’, ‘Chebychev’, ‘Cityblock’, ‘Correlation’, ‘Euclidean’, ‘Hamming’, ‘Minkowski’ and ‘Seuclidean’.

In case of the Random Forest classifier, we mainly optimized three parameters. The first one is the number of decision trees to build a random forest (NTrees), the second one is the number of attributes to select for each decision split (NVarToSample), and the last one is the minimum number of observations for each tree leaf (MinLeaf). During experiments, we set the values of these three parameters as {2^0^, 2^1^, …, 2^10^}, {1, 2, …, ‘All’} and {2^0^, 2^1^, …, 2^4^}, respectively, where ‘All’ denotes the total number of attributes.

In case of SVM, there are also two parameters which are strongly associated with its classification performance, i.e., the penalty factor *C* and the kernel function parameters [40]. The penalty factor *C* describes the tolerance for misclassified data samples, and the kernel function is used to map data into a higher dimensional space. Generally, there are four widely used kernel functions, as summarized in Table 7, where *C* is the penalty factor, *d* is the polynomial order, *c* represents the offset coefficient, and *γ* denotes the kernel bandwidth.

**Table 7:** Widely used kernel functions.

Kernel Name	Description	Parameters and Tested Values
Linear kernel	*K*(*x*_*i*_, *x*_*j*_) = *x*_*i*_ ∗ *x*_*j*_	*C*-{2^−4^, 2^−3^, …, 2^8^}
Polynomial kernel	*K*(*x*_*i*_, *x*_*j*_) = [*γ*(*x*_*i*_, *x*_*j*_)+*c*]^*d*^	*C*-{2^−4^, 2^−3^, …, 2^8^}, *γ*-{2^−4^, 2^−3^, …, 2^8^}, *c*-{0, 0.1}, *d*-{2, 3, …, 5}
RBF kernel	*K*(*x*_*i*_, *x*_*j*_) = *exp*[−*γ*|*x*_*i*_−*x*_*j*_|^2^]	*C*-{2^−4^, 2^−3^, …, 2^8^}, *γ*-{2^−4^, 2^−3^, …, 2^8^}
Sigmoid kernel	*K*(*x*_*i*_, *x*_*j*_) = *tanh*[*γ*(*x*_*i*_, *x*_*j*_)+*c*]	*C*-{2^−4^, 2^−3^, …, 2^8^}, *γ*-{2^−4^, 2^−3^, …, 2^8^}, *c*-{0, 0.1}

For each candidate parameter setting and feature selection strategy of the above three classification models, we repeated experiments for 100 time to obtain the average Accuracy, Precision and Recall. Results corresponding to the optimal classifiers were summarized in Table 8. Accordingly, we can find that SVM performs better than the other two classifiers. Specifically, the optimal SVM classifier was achieved when using the sequential forward feature selection strategy and the RBF kernel, where the values of *C* and *γ* are around 20 and 55, respectively. The reason might be that SVM is very efficient for nonlinear data distributions [41].

**Table 8:** Performances of different classification models.

Classifier	NULL			Information Gain			Sequential Forward		
	Accuracy	Precision	Recall	Accuracy	Precision	Recall	Accuracy	Precision	Recall
kNN	94.39	95.13	98.81	95.95	96.47	99.16	95.05	95.72	98.92
Random Forest	94.72	95.44	98.86	95.55	96.09	99.10	93.02	93.72	98.73
SVM	95.56	96.26	98.95	97.06	97.70	99.14	**97.57**	98.01	99.37

#### Severity assessment

**Fig 9:**
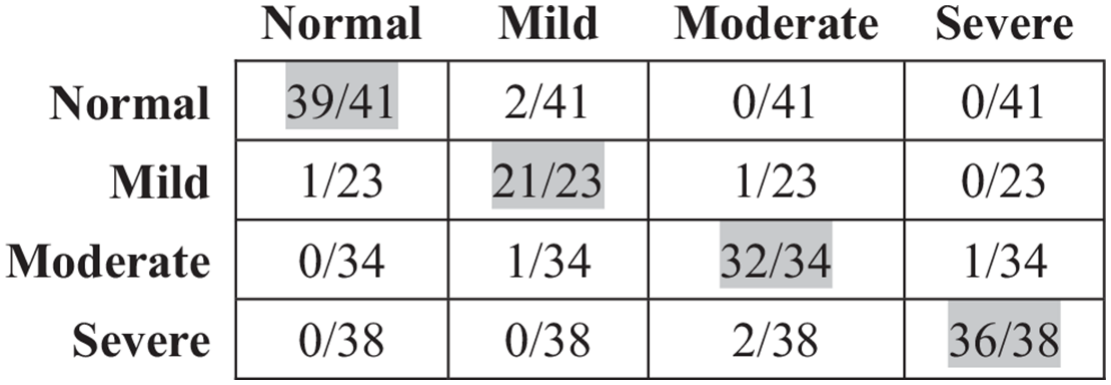
Severity assessment results.

Based on the optimal SVM classifier, SBEs and NSBEs can be identified effectively from all unequal-length RR segments with the status value of 1. Afterwards, the sleep apnea syndrome severity can be easily obtained by calculating the AHI. Fig 9 presents the severity assessment results for all the subjects in our dataset.

According to Fig 9, the accuracy of all sleep apnea syndrome severity levels and healthy subjects are satisfactory, and most errors occurred in the adjacent severity levels, which should be due to the false identification of a small number of sleep-related breathing events. Specifically, the overall accuracy (128/136 = 94.12%) firmly validated the effectiveness of the proposed methods for both sleep-related breathing event detection and sleep apnea syndrome severity assessment.

## Discussion

A novel automatic sleep apnea syndrome severity assessment approach based on the BCG signal is presented in this paper. Particularly, to record original BCG signals in an obtrusive way, we employ a sleep monitoring system in home environment. The proposed approach can distinguish each severity level effectively, i.e., healthy, mild, moderate and severe, and also overcome the problem that there are very limited differences between mild sleep apnea syndrome suffers and healthy subjects. As the detection of sleep-related breathing event is a key step for the severity assessment of sleep apnea syndrome, there are more studies on event detection than severity assessment in the literature. In order to demonstrate the performance of the proposed approach, we compared it with several most recent works on sleep-related breathing event detection, as shown in Table 9. Specifically, the sensitivity refers to the percentage of classified SBEs in all real SBEs, the specificity is the percentage of classified NSBEs in all real NSBEs, and the accuracy is the precision rate for both SBEs and NSBEs.

**Table 9:** Performance comparison among different approaches.

	A. Zaffaroni et al. [42]	B. Koley et al. [23]	J. Sole-Casals et al. [21]	J. Jin et al. [43]	Our work
**Year**	2009	2013	2014	2015	2016
**Signal/Device**	radio-frequency sensor	orinasal airflow signal	voice	MEMS sensor	BCG signal
**Segmentation method**	—	equal-length	—	—	unequal-length
**Sensitivity**	89.00	—	81.74	100.0	98.01
**Specificity**	92.00	—	82.40	85.90	91.44
**Accuracy**	91.00	96.50	82.04	—	97.57

According to Table 9, compared with existing studies, the proposed sleep-related breathing event detection and identification method achieved satisfactory performance in terms of sensitivity, specificity and accuracy, indicating that it is an efficient approach for the severity assessment of sleep apnea syndrome. Moreover, different from existing studies, on one hand, the proposed method utilized the BCG signal, which can be easily obtained and affordable in home environment in a non-invasive manner. On the other hand and more importantly, all the severity levels can be accurately identified with the proposed method.

We further explain the reason why sleep apnea syndrome (a respiration dynamics-related disease) can be assessed based on cardiovascular activities, to be more specific, features extracted from the RR interval time series. On one hand, sleep apnea syndrome can cause intermittent cardiac hypoxia, and one of the most prominent features of hypoxia is mitochondrial dysfunction, which affects cells and cellular components and induces increased production of reactive oxygen species. Reactive oxygen species will further lead to enhanced oxidative stress, which can induce sympathetic hyper-activation. On the other hand, the heart rate is controlled by the autonomic nervous system, which consists of the sympathetic nervous system and the parasympathetic nervous system [44]. In other words, the hyper-activation of sympathetic nerve will influence heart rate. Thereby, sleep apnea syndrome can be diagnosed by analyzing the variability of heart rate [45].

However, there are still several limitations in our current study. First of all, the experiment dataset is not sufficient enough, and the number of subjects for each severity level is limited. Therefore, more data is needed for clinical validation in the future. In addition, we only have one night’s sleep data for each of the subjects in our experiment, which is not enough for personalized modeling.

## Conclusion

In this paper, we present an automatic severity assessment approach for sleep apnea syndrome based on the BCG signal, which is recorded using a non-invasive sleep monitoring system in home environment. The proposed approach can provide an estimation of the apnea hypopnoea index by detecting and identifying the sleep-related breathing events, which mainly consists of three stages, i.e., data preprocessing, sleep-related breathing event detection and severity evaluation. In the data preprocessing stage, the wavelet decomposition and overlapped sliding window method was applied, based on which the time series of RR intervals could be obtained and corrected. In the sleep-related breathing event detection stage, we proposed the Physio_ICSS algorithm which can avoid the segmentation issue of sleep-related breathing events. In the severity evaluation stage, we identified sleep-related breathing events by extracting features from the corresponding RR fragments. Meanwhile, we had tested various classifiers and different classifier parameters to optimize the identification performance. Compared with existing studies, experimental results validated the effectiveness of the proposed approach.

As existing medical system can hardly provide non-invasive and continuous health monitoring in our daily life, this work can be regarded as a significant attempt in the health care field, which can help to estimate sleep apnea syndrome and other cardiac diseases in home environments.

## Supporting information

S1 Algorithm The Physio_ICSS algorithm. Detailed description to the Physio_ICSS Algorithm.
(PDF)

## References

[1] Young T, Finn L, Austin D, Peterson A. Menopausal status and sleep-disordered breathing in the Wisconsin sleep cohort study. American Journal of Respiratory and Critical Care Medicine. 2003; 167:1181–1185. 10.1164/rccm.200209-1055OC 12615621

[2] Kim J, In K, Kim J, You S, Kang K, Shim J, et al Prevalence of Sleep-disordered Breathing in Middle-aged Korean Men and Women. American Journal of Respiratory and Critical Care Medicine. 2004; 170(10):1108–1113. 10.1164/rccm.200404-519OC 15347562

[3] Lam B, Lam DCL, Ip MSM. Obstructive sleep apnoea in Asia. The International Journal of Tuberculosis and Lung Disease. 2007; 11(1):2–11. 17217123

[4] Malhotra A, White DP. Obstructive sleep apnea. Lancet. 2002; 360(9328):237–45. 10.1016/S0140-6736(02)09464-3 12133673

[5] Goel N, Rao H, Durmer JS, Dinges DF. Neurocognitive consequences of sleep deprivation. Seminars in Neurology. 2005; 25(1):117–29. 10.1055/s-2005-867080 15798944

[6] Redline S, Tosteson T, Boucher MA, Millman RP. Measurement of sleep-related breathing disturbances in epidemiologic studies: assessment of the validity and reproducibility of a portable monitoring device. Chest. 1991; 100(5):1281–1286. 10.1378/chest.100.5.1281 1935282

[7] Guilleminault C, Tilkian A, Dement WC. The Sleep Apnea Syndromes. Annual Review of Medicine. 1976; 27(1):465–484. 10.1146/annurev.me.27.020176.002341 180875

[8] Flemons WW, Buysse D, Redline S, Oack A, Strohl K, Wheatley J, Young T, Douglas N, Levy P, McNicolas W, Fleetham J, White D, Schmidt-Nowarra W, Carley D, Romaniuk J. Sleep-related breathing disorders in adults. Sleep Journal. 1999; 22(5):667–689.

[9] Bloch K. Polysomnography: a systematic review. Technology and health care. 1997; 5:285–305. 9429270

[10] Marcos J, Hornero R, Nabney T, Alvarez D, Campo F. Analysis of Nocturnal Oxygen Saturation Recordings using Kernel Entropy to Assist in Sleep Apnea-Hypopnea Diagnosis. In: Proceedings of the 33rd Annual International Conference of the IEEE Engineering in Medicine and Biology Society; 2011. p. 1745–1748. 10.1109/IEMBS.2011.6090499 22254664

[11] Koley B, Dey D. Real-Time Adaptive Apnea and Hypopnea Event Detection Methodology for Portable Sleep Apnea Monitoring Devices. IEEE Transactions on Biomedical Engineering. 2013; 60(12):3354–3363. 10.1109/TBME.2013.2282337 24058010

[12] Deuchar DC. Ballistocardiography. British Heart Journal. 1967; 29(3):285–288. 10.1136/hrt.29.3.285 5337199 PMC459147

[13] Giovangrandi L, Inan OT, Wiard RM, Etemadi M, Kovacs GTA. Ballistocardiography: A method worth revisiting. In: Proceedings of the 2011 Annual International Conference of the IEEE Engineering in Medicine and Biology Society; 2011. p. 4279–4282. 10.1109/IEMBS.2011.6091062 PMC4274997 22255285

[14] Martínez-Vargas JD, Sepulveda-Cano LM, Travieso-Gonzalez C, Castellanos-Dominguez G. Detection of Obstructive Sleep Apnoea Using Dynamic Filter-banked Features. Expert Syst Appl. 2012; 39(10):9118–9128. 10.1016/j.eswa.2012.02.043

[15] Alvarez D, Hornero R, Marcos JV, del Campo F. Multivariate Analysis of Blood Oxygen Saturation Recordings in Obstructive Sleep Apnea Diagnosis. IEEE Transactions on Biomedical Engineering. 2010; 57(12):2816–2824. 10.1109/TBME.2010.2056924 20624698

[16] Zhao W, Ni H, Zhou X, Song Y, Wang T. Identifying sleep apnea syndrome using heart rate and breathing effort variation analysis based on ballistocardiography. In: Proceedings of the 2015 37th Annual International Conference of the IEEE Engineering in Medicine and Biology Society (EMBC); 2015. p. 4536–4539. 10.1109/EMBC.2015.7319403 26737303

[17] Migliorini M, Bianchi AM, Nistico D, Kortelainen J, Arce-Santana E, Cerutti S, et al. Automatic sleep staging based on ballistocardiographic signals recorded through bed sensors. In: Proceedings of the 2010 Annual International Conference of the IEEE Engineering in Medicine and Biology; 2010. p. 3273–3276. 10.1109/IEMBS.2010.5627217 21096612

[18] Casals J, Munteanu C, Martin O, Barbe F, Queipo C, Amilibia J, Cantolla J. Detection of Severe Obstructive Sleep Apnea through Voice Analysis. Applied Soft Computing. 2014; 23(2014):346–354. 10.1016/j.asoc.2014.06.017

[19] Xie B, Minn H. Real-Time Sleep Apnea Detection by Classifier Combination. IEEE Transactions on Information Technology in Biomedicine. 2012; 16(3):469–477. 10.1109/TITB.2012.2188299 22353404

[20] Golemati S, Zourou V, Nikita KS. Comparison of entropy measures for estimating severity of obstructive sleep apnea from overnight pulse oximetry data. In: Proceedings of the 10th IEEE International Conference on Information Technology and Applications in Biomedicine; 2010. p. 1–4.

[21] Sole-Casals J, Munteanu C, Martin OC, Barbe F, Queipo C, Amilibia J, et al Detection of severe obstructive sleep apnea through voice analysis. Applied Soft Computing. 2014; 23:346–354. 10.1016/j.asoc.2014.06.017

[22] Bsoul M, Minn H, Tamil L. Apnea MedAssist: Real-time Sleep Apnea Monitor Using Single-Lead ECG. IEEE Transactions on Information Technology in Biomedicine. 2011; 15(3):416–427. 10.1109/TITB.2010.2087386 20952340

[23] Koley BL, Dey D. Real-Time Adaptive Apnea and Hypopnea Event Detection Methodology for Portable Sleep Apnea Monitoring Devices. IEEE Transactions on Biomedical Engineering. 2013; 60(12):3354–3363. 10.1109/TBME.2013.2282337 24058010

[24] Chen L, Zhang X, Song C. An Automatic Screening Approach for Obstructive Sleep Apnea Diagnosis Based on Single-Lead Electrocardiogram. IEEE Transactions on Automation Science and Engineering. 2015; 12(1):106–115. 10.1109/TASE.2014.2345667

[25] Chen L, Zhang X, Song C. A severity measurement system for obstructive sleep apnea discrimination using a single ECG signal. In: Proceedings of the 2013 IEEE International Conference on Automation Science and Engineering (CASE); 2013. p. 1–6.

[26] Li W, Wang R, Huang D. Assessment of Micro-movement Sensitive Mattress Sleep Monitoring System (RS611) in the detection of obstructive sleep apnea hypopnea syndrome. Chinese Journal of Gerontology. 2015; 35(5):1160–1162.

[27] The AASM Manual for the Scoring of Sleep and Associated Events: Rules, Terminology and Technical Specification; 2007.

[28] Chouchou F, Pichot V, Barthelemy J, Bastuji H, Roche F. Cardiac Sympathetic Modulation in Response to Apneas/Hypopneas through Heart Rate Variability Analysis. PLoS ONE. 2014; 9:e86434 10.1371/journal.pone.0086434 24466093 PMC3899280

[29] Keissar K, Davrath L, Akselrod S. Coherence analysis between respiration and heart rate variability using continuous Wavelet transform. Phil Trans R Soc A. 2009; 2009(367):1393–1406. 10.1098/rsta.2008.0273 19324715

[30] Chen SW. A wavelet-based heart rate variability analysis for the study of nonsustained ventricular tachycardia. IEEE Transactions on Biomedical Engineering. 2002; 49(7):736–742. 10.1109/TBME.2002.1010859 12083310

[31] Ewing BT, Malik F. Re-examining the asymmetric predictability of conditional variances: The role of sudden changes in variance. Journal of Banking & Finance. 2005; 29(10):2655–2673. 10.1016/j.jbankfin.2004.10.002

[32] Rapach DE, Strauss JK. Structural breaks and GARCH models of exchange rate volatility. Journal of Applied Econometrics. 2008; 23(1):65–90. 10.1002/jae.976

[33] Ravenswaaij C, Kollee L, Hopman J, Stoelinga G, Geijn H. Heart Rate Variability. Annals of Internal Medicine. 1993; 118(6):436–447. 10.7326/0003-4819-118-6-199303150-00008 8439119

[34] Kasai T, Bradley TD. Obstructive Sleep Apnea and Heart Failure: Pathophysiologic and Therapeutic Implications. Journal of the American College of Cardiology. 2011; 57(2):119–127. 10.1016/j.jacc.2010.08.627 21211682

[35] Bianchi AM, Mendez MO. Methods for heart rate variability analysis during sleep. In: Proceedings of the 35th Annual International Conference of the IEEE Engineering in Medicine and Biology Society (EMBC); 2013. p. 6579–6582. 10.1109/EMBC.2013.6611063 24111250

[36] Boardman A, Schlindwein FS, Rocha AP, Leite A. A study on the optimum order of autoregressive models for heart rate variability. Physiological Measurement. 2002; 23(2):325–336. 10.1088/0967-3334/23/2/308 12051304

[37] Kantelhardt JW, Koscielny-Bunde E, Rego HHA, Havlin S, Bunde A. Detecting long-range correlations with detrended fluctuation analysis. Physica A: Statistical Mechanics and its Applications. 2001; 295(3):441–454. 10.1016/S0378-4371(01)00144-3

[38] Pincus SM. Approximate entropy as a measure of system complexity. Proceedings of the National Academy of Sciences. 1991; 88(6):2297–2301. 10.1073/pnas.88.6.2297 PMC51218 11607165

[39] Xiao M, Yan H, Yang X, Li Y, Zhu R. Multiscale entropy based analysis of HRV during sleep. In: Proceedings of the 5th International Conference on Biomedical Engineering and Informatics (BMEI); 2012. p. 558–562.

[40] Vapnik V, Chapelle O. Bounds on Error Expectation for Support Vector Machines. Neural Computation. 2000; 12(9):2013–2036. 10.1162/089976600300015042 10976137

[41] Hearst MA, Dumais ST, Osman E, Platt J, Scholkopf B. Support vector machines. IEEE Intelligent Systems and their Applications. 1998; 13(4):18–28. 10.1109/5254.708428

[42] Zaffaroni A, de Chazal P, Heneghan C, Boyle P, Mppm PR, McNicholas WT. SleepMinder: An innovative contact-free device for the estimation of the apnoea-hypopnoea index. In: Proceedings of the 2009 Annual International Conference of the IEEE Engineering in Medicine and Biology Society; 2009. p. 7091–7094. 10.1109/IEMBS.2009.5332909 19963942

[43] Jin J, Sanchez-Sinencio E. A Home Sleep Apnea Screening Device With Time-Domain Signal Processing and Autonomous Scoring Capability. IEEE Transactions on Biomedical Circuits and Systems. 2015; 9(1):96–104. 10.1109/TBCAS.2014.2314301 25486649

[44] Lavie L, Lavie P. Molecular mechanisms of cardiovascular disease in OSAHS: the oxidative stress link. European Respiratory Journal. 2009; 33(6):1467–1484. 10.1183/09031936.00086608 19483049

[45] Acharya U, Joseph K, Kannathal N, Lim C, Suri J. Heart rate variability: a review. Med Bio Eng Comput. 2006; 44:1031–1051. 10.1007/s11517-006-0119-0 17111118

