# Supplementary material for: Assessing the severity of sleep apnea syndrome based on ballistocardiogram
Source: PLoS One. 2017 Apr 26;12(4):e0175351. doi: 10.1371/journal.pone.0175351 (PMC5405918; doi:10.1371/journal.pone.0175351)
Supplement: S1 Algorithm — Detailed description to the Physio_ICSS Algorithm. (PDF) [file pone.0175351.s001.pdf]

Supporting Information

The Physio_ICSS Algorithm

Based on the concept of iterative cumulative sum of squares, we propose a sleep-related breathing event detection algorithm named Physio_ICSS, which is able to detect sudden structural changes in long time series [1,2]. Specifically, given a time series $\{y_t\}$ which follows the normal distribution with mean μ and constant variance σ^2, $t = 1, 2, 3, \ldots,$ T , its residual series $\{x_t\}$ can be represented as:

$$x_t = (y_t - \frac{1}{t}\sum_{i=0}^{t} y_i)/\sqrt{\frac{S_y^2(t+1)}{t}}, \quad (1)$$

where S_y^2 is the estimate of $\{y_t\}$'s variance. According to existing studies [1,2], $\{x_t\}$ also follows the normal distribution. To detect sudden structural changes of $\{y_t\}$, the original ICSS algorithm proposed to use the centered (and normalized) cumulative sum of squares which is defined as:

$$D_t = \frac{C_t}{C_T} - \frac{t}{T}, \quad (2)$$

where $C_t = \sum_{i=1}^{t} x_i^2$, $t = 1, 2, 3, ..., T$, is the cumulative sum of squares of x_t.

When there is a sudden change in variance, the value of D_t will exceed some specified boundaries with high probability. These boundaries can be obtained from the asymptotic distribution of D_t assuming constant variance. According to [3], we can search for a variance change point via $max_t|D_t|$. In particular, let k^* be the value of t where $max_t|D_t|$ is attained, if this maximum absolute value exceeds a pre-determined boundary, then we may conclude that there is a change point near k^* and take k^* as an estimate of the change point. Specifically, if the residual series $\{x_t\}$ is a constant sequence, i.e., $x_1 = x_2 = ... = x_t = ... = x_T$, the value of D_t would be 0, and there are no sudden change points in $\{y_t\}$.

To further test whether a change point is of certain significance, Inclan and Tiao [3] proposed the use of a statistics named IT, which is defined as follows:

$$IT = \sup_t |\sqrt{\frac{T}{2D_t}}|, \quad (3)$$

and its asymptotic distribution is given by:

$$IT \Rightarrow \sup_r |W^*(r)|, \quad (4)$$

where $W^*(r) \equiv W(r) - rW(1)$ is a Brownian Bridge, $W(r)$ is a standard Brownian motion, and $\Rightarrow$ stands for weak convergence of the associated probability measures. For a given sample, if IT is greater than a specified critical value θ, i.e., $\max(\sqrt{\frac{T}{2D_t}}) > \theta$, then there is a significant change point in the time series, and vice versa.

However, due to the inherent complexity and variability of human bodies, the physiological data usually is conditional variance heteroscedastic, and its residual series can not satisfy the constraint of independent normal distribution. Moreover, when ICSS is used to analyze physiological data, the IT statistics usually would be over-estimated,

resulting in many fake change points. To this end, Sansó et al. [4] revised the definition of the IT statistics as follows:

$$\kappa_2 = \sup_t |T^{-1/2} G_t|, \tag{5}$$

where

$$G_t = \widehat{\omega}_4^{-\frac{1}{2}} (C_t - \frac{t}{T} C_T), \tag{6}$$

and $\widehat{\omega}_4$ is a consistent estimator of ω_4, which can be calculated as follows:

$$\begin{aligned} \widehat{\omega}_4 = \; & \tfrac{1}{T} \textstyle\sum_{t=1}^{T} (x_t^2 - \widehat{\sigma}^2)^2 + \\ & \tfrac{2}{T} \textstyle\sum_{l=1}^{m} w(l,m) \sum_{t=l+1}^{T} (x_t^2 - \widehat{\sigma}^2)(x_{t-l}^2 - \widehat{\sigma}^2), \end{aligned} \tag{7}$$

where $w(l,m)$ is a lag window defined as $w(l,m) = 1 - \frac{l}{m+1}$ or the quadratic spectral. This estimator depends on the selection of the bandwidth m, which can be chosen using an automatic procedure as proposed in [5].

Based on the above definitions and formulas, we can conclude that both the original and the revised ICSS algorithms need a plenty of iterative computations. In case that there is a large amount of data (e.g., numerous users' high frequency sampling BCG data of the whole night), the computational efficiency will be severely declined. Thereby, we propose to improve the ICSS algorithm's performance by taking into account the practical factors of sleep-related breathing events as aforementioned, and put forward the Physio_ICSS algorithm, as described in Algorithm S1.

At the beginning, given a time series $[t_1, t_2]$ longer than 10 seconds, we try to obtain its first and last structural change points (i.e., k_{first} and k_{last}) by calculating $M_t[t_1 : t_2]$ based on the κ_2 statistics (lines 4-18). Afterwards, if the detected k_{first} and k_{last} are two distinct change points (line 18), they will be recorded in vector P and the algorithm will iteratively obtain all the change points in the new time interval [k_{first} + 1, k_{last} - 1] (lines 20-23). Furthermore, for each point P_j in vector P, we check whether it is a true change point (lines 25-29). Finally, we label the intervals between two adjacent change points using a status array S to represent whether there might be sleep-related breathing events within the corresponding time interval or not, where 1 means *yes*, and 0 stands for *no* (lines 31-37).

References

1. Ewing BT, Malik F. Re-examining the asymmetric predictability of conditional variances: The role of sudden changes in variance. Journal of Banking & Finance. 2005; 29(10):2655–2673.

2. Rapach DE, Strauss JK. Structural breaks and GARCH models of exchange rate volatility. Journal of Applied Econometrics. 2008; 23(1):65–90.

3. Inclan C, Tiao GC. Use of Cumulative Sums of Squares for Retrospective Detection of Changes of Variance. Journal of the American Statistical Association. 1994; 89(427):913–923.

4. Sanso A, Arago V, Carrion-i Silvestre J. Testing for Changes in the Unconditional Variance of Financial Time Series. Revista de EConomia FinanCiera. 2004; 4:32–51.

5. Newey WK, West KD. Automatic Lag Selection in Covariance Matrix Estimation. Review of Economic Studies. 1994; 61:631–653.

Algorithm 1 The Physio_ICSS Algorithm

Require:

- X, the raw time series

Ensure:

- PF, the corresponding sequence of points where structural changes happen
- S, the status array that labels whether a certain segment might contain sleep-related breathing events

1: $t_1 \leftarrow 1, t_2 \leftarrow T$
2: $k_first \leftarrow t_1, k_last \leftarrow t_1$;
3: **repeat**
4: calculate each $G_t[t_1 : t_2]$ based on formula (6)
5: $M_t[t_1 : t_2] \leftarrow \max(\frac{|G_t[t_1:t_2]|}{\sqrt{t_2 - t_1 + 1}})$
6: **if** $M_t[t_1 : t_2] > G^*$ (G^* is the threshold for a certain confidence level) **then**
7: $t_2 \leftarrow k^*[t_1 : t_2]$ (k^* is the point that $G_k[t_1 : t_2]$ achieves the maximum value)
8: **if** $t_1 = 1$ **then**
9: $k_{first} \leftarrow t_2, t_1 \leftarrow k_{first} + 1, t_2 \leftarrow T$ (k_{first} is the first structural change point, and $[t_1, t_2]$ (i.e., $[k_{first} + 1, T]$) is the new target time interval)
10: **else**
11: $t_1 \leftarrow t_2 + 1, t_2 \leftarrow T$
12: **end if**
13: **else**
14: $k_{last} \leftarrow max(t_1 - 1, 1)$
15: break
16: **end if**
17: **until** $t_2 - t_1 \leq 10s$
18: **if** $k_{first} < k_{last}$ **then**
19: $t_1 \leftarrow k_{first} + 1, t_2 \leftarrow k_{last} - 1$
20: **repeat**
21: put k_{first} and k_{last} into vector P in ascending order
22: do lines 4-18
23: **until** $k_{first} \geq k_{last}$
24: **end if**
25: **for** each $P_j \in P$ **do**
26: **if** $M[P_{j-1} + 1 : P_j] \leq G^*$ **then**
27: eliminate P_j (eliminate the fake change points)
28: **end if**
29: **end for**
30: put the remaining P_j into vector PF;
31: **for** each $PF_j, PF_{j+1} \in PF$ **do**
32: **if** $PF_{j+1} - PF_j \geq 10s$ **then**
33: $S_j = 1$
34: **else**
35: $S_j = 0$
36: **end if**
37: **end for**
